# A young man with DiGeorge syndrome and tachycardia

**DOI:** 10.1007/s12471-023-01780-5

**Published:** 2023-05-05

**Authors:** Nicolas Bradt, Leonie Franceus, Alice Fouckova, Becker Alzand

**Affiliations:** 1 Department of Cardiology, AZ Glorieux, Ronse, Belgium; 2 Department of Internal Medicine, UZ Ghent, Ghent, Belgium; 3 Department of Medicine, University of Ghent, Ghent, Belgium; 4 Department of Internal Medicine, AZ Glorieux, Ronse, Belgium

A 24-year-old man presented to the emergency department complaining of palpitations. The symptoms had lasted for up to 4 h. He is known to have a 22q11.2 microdeletion (DiGeorge syndrome) as well as tetralogy of Fallot, for which surgical correction was performed in 1998. The patient has a mild cognitive impairment but no other clinical features of the DiGeorge syndrome. Furthermore, in 2017 the patient underwent a pulmonary homograft due to valve insufficiency combined with closure of a patent foramen ovale. Previous electrocardiograms (ECGs) showed a complete right bundle branch block with a similar QRS morphology to the current ECG during tachycardia. His only current medication is acetylsalicylic acid 80 mg once daily. The initial ECG (Fig. 1) was performed on his arrival at the emergency department. The second ECG (Fig. 2) was performed after intravenous administration of 12 mg adenosine.

**Fig. 1:**
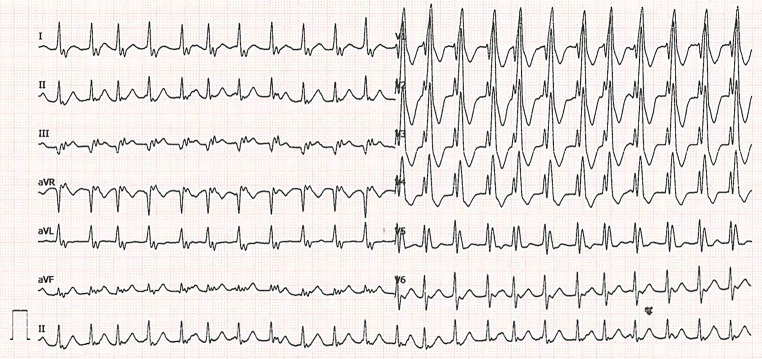
12-lead resting ECG performed at the emergency department

**Fig. 2:**
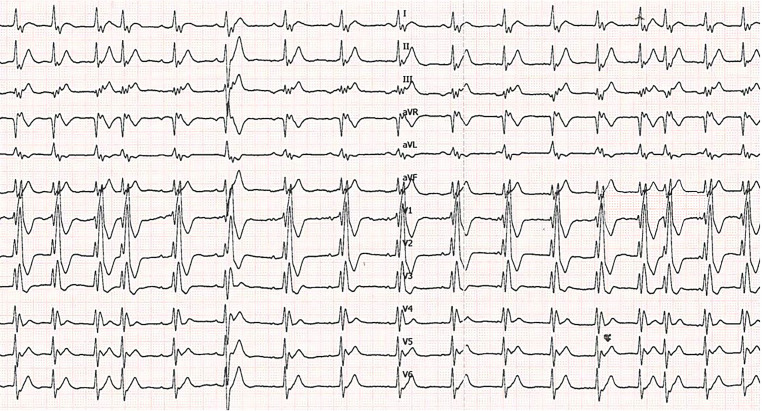
12-lead resting ECG performed after the administration of 12 mg adenosine

What is your diagnosis and how do you explain the slight irregularity?

## Answer

You will find the answer elsewhere in this issue.

